# Loss of Ufsp1 does not cause major changes at the neuromuscular junction

**DOI:** 10.1371/journal.pone.0328690

**Published:** 2025-08-01

**Authors:** Cristofer Calvo, Coalesco Smith, Taejeong Song, Fabian Montecino-Morales, Sakthivel Sadayappan, Douglas P. Millay, Minchul Kim

**Affiliations:** 1 Division of Molecular Cardiovascular Biology, Cincinnati Children’s Hospital Medical Center, Cincinnati, Ohio, United States of America; 2 Universite´ de Strasbourg, Strasbourg, France; 3 CNRS, Illkirch, France; 4 INSERM, Illkirch, France; 5 IGBMC, Institut de Génétique et de Biologie Mole´ culaire et Cellulaire, Illkirch, France; 6 Center for Cardiovascular Research, Department of Internal Medicine, University of Cincinnati College of Medicine, Cincinnati, Ohio, United States of America; 7 Department of Pediatrics, University of Cincinnati College of Medicine, Cincinnati, Ohio, United States of America; Fujita Health University: Fujita Ika Daigaku, JAPAN

## Abstract

UFMylation is a Ubiquitin-like post-translational modification involved in myriad of cellular processes. Enzymes involved in this pathway, including ligases and UFM1-specific proteases, are essential for development and homeostasis. Our previous transcriptomic analyses identified an enrichment of Ufsp1 at the neuromuscular junction of skeletal muscle cells. Ufsp1, one of the two UFM1 proteases, had been considered a pseudogene due to truncation of its catalytic domain in several species, including humans. However, recent findings revealed that Ufsp1 is translated from a non-canonical start codon in humans, yielding a catalytically active enzyme. This discovery has revived interest in studying Ufsp1’s role in vivo. We generated two mutant mouse models, one with a point mutation abolishing catalytic activity and another with complete knockout of the gene. Unlike other UFMylation pathway enzymes, both Ufsp1 mutants were born in normal ratios and did not exhibit gross phenotypic abnormalities. Despite the enrichment of Ufsp1 at neuromuscular junctions, only mild structural alterations of this synapse were detected, which did not impact overall muscle function. Our findings indicate that Ufsp1 is dispensable for normal development and homeostasis in mice, but further exploration of its function is needed in pathological conditions.

## Introduction

A defining feature of skeletal muscle fibers (myofibers) is their syncytial nature, where hundreds to thousands of nuclei share a common cytoplasm, allowing for highly coordinated gene expression and cellular function [1,2]. Within this shared cytoplasm, one compartment in myofibers that requires unique transcriptional control is the neuromuscular junction (NMJ), which is a specialized muscle membrane domain positioned at the center of myofibers where the axon terminals of motor neurons establish synaptic contact [3,4]. In the mouse, this specialized membrane contains a high density of acetylcholine receptors (AChRs, ~ 10000/μm^2^) that form clusters with a pretzel-like morphology [5]. Upon neural stimulation, acetylcholine is released from the presynaptic terminal, binding to AChR clusters, thereby triggering action potentials and myofiber contraction. Mouse genetic studies and characterization of neuromuscular pathologies have demonstrated that Agrin/MuSK signaling is essential for the establishment and maintenance of AChR clusters and overall NMJ integrity and function [6–11].

Myofibers exhibit a compartmentalized transcription of canonical NMJ genes underneath the AChR clusters [12,13]. We and others have previously performed single-nucleus RNA-Sequencing (snRNA-Seq) analysis of wild-type skeletal muscle tissues [14–16], uncovering a set of genes selectively enriched at the NMJ, many of which have unknown functions in myofibers. This observation suggests that characterization of these genes may uncover novel insights into NMJ development, maintenance and remodeling in healthy or diseased states. Our snRNA-Seq found *Ufsp1* as a top enriched gene of NMJ-associated myonuclei and the depletion of *Ufsp1* increases AChR clustering *in vitro* [15], suggesting a potential role as modulator of NMJ formation.

*Ufsp1* encodes for an enzyme (Ufsp1) involved in the UFMylation pathway, a type of Ubiquitin-like post-translational modification that has recently attracted significant attention because it regulates various biological processes, including ER membrane homeostasis, translation fidelity, DNA damage repair, response to viral infection, among others [17–22]. Similar to ubiquitination, UFMylation involves a cascade of enzymatic actions catalyzed by E1 (UBA5), E2 (UFC1), and E3 (UFL1) proteins to attach UFM1 onto lysine residues of target substrates [23,24]. UFMylation can also be reversed by UFSP proteins (UFSP1 and UFSP2), which function similarly to de-ubiquitinases. Additionally, UFSP proteins are essential for the proteolytic maturation of pro-UFM1 into UFM1, suggesting that UFSP proteins can promote or inhibit UFMylation, likely depending on the substrate and biological context. Furthermore, i*n vivo* genetic deletion of UFMylation enzymes, including *Ufsp2*, have demonstrated an essential role of UFMylation in mouse embryonic development and tissue homeostasis ( [25–28] and https://www.mousephenotype.org/about-impc/about-ikmc/eucomm/).

Human UFSP1 has long been considered as a pseudogene because its catalytic domain has been deleted. However, recent studies discovered that human UFSP1 is translated from a non-canonical upstream codon in cultured human cell lines and produces a fully catalytically active protein [29,30]. Given the general essentiality of UFMylation enzymes in development, the potential role of *Ufsp1* in NMJ biology and the recent implication of the human UFSP1 as a bona fide enzyme, it became apparent to analyze the function of *Ufsp1 in vivo*.

## Results

**Fig 1:**
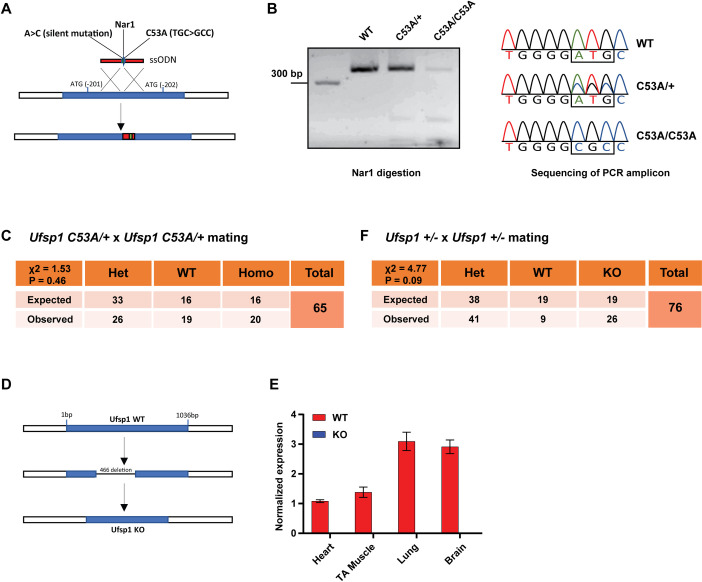
Genetic deletion of Ufsp1 or disruption of enzymatic activity is compatible with normal mouse development. (A) Description of Ufsp1 C53A targeting strategy. (B) Representative agarose gel image after PCR and Nar1 digestion from indicate genotypes. (C) Sequencing of PCR amplicon verifies the targeted mutation. (D) Ufsp1 C53A mutants are born in normal mendelian ratio after heterozygote mating. The statistical significance of difference between expected and observed number of pups of each genotype was quantified by chi-square test. (D) Description of Ufsp1 KO targeting strategy. (E) RT-qPCR of indicated tissues from WT and KO mice confirms the loss of Ufsp1 in the mutants (n = 3). Error bars indicate S.E.M. (F) Ufsp1 KO mutants are born in normal mendelian ratio after heterozygote mating. The statistical test is the same as (D).

To test the requirement Ufsp1’s enzymatic activity in development, we generated a C53A knock-in mutant mouse line (Fig 1A). Cysteine 53 is a core part of catalytic pocket and its mutation to Alanine abolishes the activity of Ufsp1 [31,32]. Restriction enzyme digestion as well as sequencing of the PCR amplicon around C53A site confirmed the mutation (Fig 1B). Mating between *Ufsp1*^*C53A/+*^ heterozygotes resulted in homozygous mutants at the expected Mendelian ratio (Fig 1C). Furthermore, the C53A homozygous mutants did not display gross abnormalities throughout the observation period of up to 1 year.

To explore potential catalytic activity-independent functions of Ufsp1, we generated a full knockout mouse model using CRISPR/Cas9 to delete ~466 bp of the Ufsp1 gene (notably, Ufsp1 is a single exon gene) (Fig 1D). RT-qPCR analysis across multiple tissues (skeletal muscle, lung, heart, and brain) confirmed the complete absence of Ufsp1 expression (Fig 1E). Similar to Ufsp1 C53A mutants, mating of *Ufsp1*^*+/-*^ heterozygotes yielded homozygous mutants in the expected Mendelian ratio (Fig 1F), and no gross phenotypic defects were observed throughout a year of observation.

**Fig 2:**
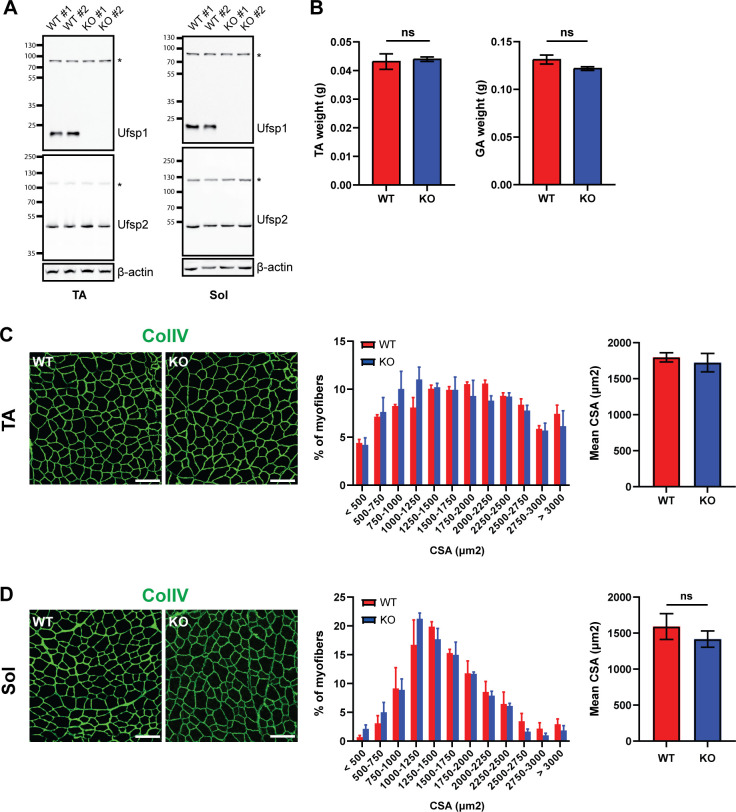
Normal skeletal muscle architecture in the absence of Ufsp1. (A) Lysates of tibilias anterior (TA) and soleus (Sol) muscles of WT and Ufsp1 KO mice were analyzed by Western blotting for the indicated targets. On the left of gels are molecular weights in kilo-Dalton (kD). *, non-specific bands. (B) Tibialis anterior (TA) and gastrocnemius (GA) muscle weights were measured (n = 3). (C-D) Cross-sectional areas (CSA) of TA and Soleus muscles were quantified based on ColV immunohistochemistry (n = 3). Scale bar, 100 µm. Error bars indicate S.E.M. Two-tailed, unpaired student’s t-test. ns, non-significant.

Given the enriched expression of *Ufsp1* at the NMJ, we next investigated the impact of *Ufsp1* loss in muscle tissues. We first verified the absence of Ufsp1 protein in the tibialis anterior (TA) and soleus (Sol) muscles in the Ufsp1 KO mutants, whereas the level of Ufsp2 was unchanged (Fig 2A). At 3 months of age, the weights of TA and gastrocnemius (GA) muscles did not show significant differences between wild-type (WT) littermate controls and Ufsp1 KO or C53A mutants (Fig 2B and S1A Fig). Additionally, the cross-sectional areas of myofibers were comparable between WT and mutant mice in both TA (fast-twitch) and Sol (slow-twitch) muscles (Fig 2C, 2D, S1B Fig and S1C Fig). These results indicate that the absence of Ufsp1 or its enzymatic activity does not result in major structural abnormalities in skeletal muscle.

We next performed more in-depth analysis of skeletal muscle focusing on the KO model. Examination of fiber type composition revealed similar distribution in both TA and Sol muscles (S2A Fig and S2B Fig). We also examined whether loss of *Ufsp1* results in degenerative/regenerative responses by assessing the frequency Pax7^+^ satellite cells, which was comparable between genotypes (S2C Fig and S2D Fig). Accordingly, the percentage of centralized myonuclei remained minimal in both WT and KO mice (S2C Fig and S2D Fig). Finally, Gomori trichrome staining, which detects fibrosis and mitochondrial abnormalities, revealed no differences (S2E Fig), consistent with the unchanged fiber type composition.

**Fig 3:**
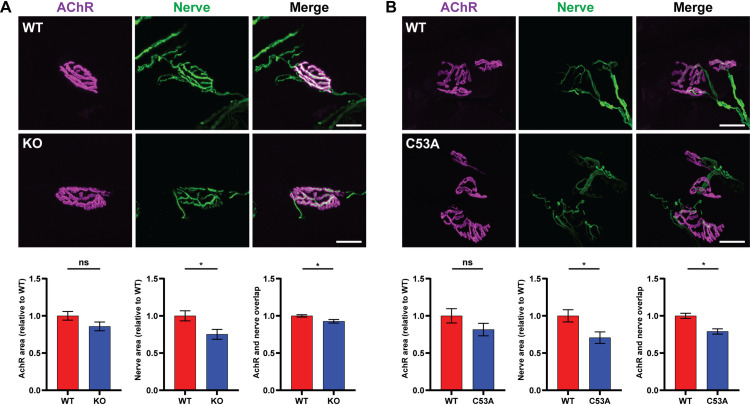
Loss of Ufsp1 causes alterations in nerve-AchR interactions at the NMJ. (A) Representative images of BTX (magenta) and motor neuron axons plus nerve terminals (neurofilament and synapsin; NF/Syn in green) co-staining in Ufsp1 KO mutants and their corresponding WT littermates in TA muscles. AchR area (BTX+), nerve area (NF/Syn+), and innervation index (overlap between BTX and NF/Syn) were quantified from the immunostaining images (n = 29 for both WT and KO total, collected from 3 mice for each genotype, at least 9 independent NMJs analyzed per individual). **(B)** Same analyses as (A) for Ufsp1 C53A mutants and WT controls (n = 23 for WT and n = 26 for C53A total, collected from 3 mice for each genotype, at least 7–8 independent NMJs analyzed per individual). Scale bar, 10 µm. Error bars indicate S.E.M. *, p-value < 0.05. ns, non-significant. Two-tailed, unpaired t-test.

Given that *Ufsp1* loss does not lead to general muscle defects, we next evaluated NMJ morphology by co-staining AChR (BTX) and motor neuron axons and nerve terminals (neurofilament and synapsin; NF/Syn). While WT and KO mutants had similar area of BTX-positive staining, that of NF/Syn-positive staining was decreased in the *Ufsp1* mutants (Fig 3A). Furthermore, the degree of NMJ innervation, defined by overlap between AChR and motor neuron axons, was significantly reduced in the KO mutants. Similar results were observed in the C53A mutants (Fig 3B). These findings suggest that the absence of *Ufsp1* or its enzymatic activity may induce a retrograde signal from the muscle to the nerve terminals, resulting in a small decrease of the presynaptic area. However, we cannot completely rule out the possibility of a motor neuron-intrinsic function of Ufsp1. Also, we note that our current analysis of NMJ morphology is limited to fast-twitch type muscles, and it would be worth testing slow-twitch muscles, where NMJ dynamics differ, in the future.

Despite these mild structural changes in the NMJ, grip strength as well as precise *in situ* measurements of contractile indexes showed no functional impairments in KO mutants (S3A Fig and S3B Fig). Additionally, RT-qPCR of TA and GA muscles did not detect significant changes in representative neurotrophic factors (*Bdnf*, *Ngf* and *Nt3*) or major NMJ-associated genes (AChR subunits, *Colq* and *Musk*) (S3C Fig).

## Discussion

UFMylation pathway has recently emerged as a versatile and critical post-translational modification for normal development and homeostatic maintenance of multiple organs. Our results show that *Ufsp1* is dispensable and the mutants are grossly normal. This is in stark contrast with other UFMylation pathway genes where the mutants show severe multi-organ phenotypes. Therefore, it appears that the major operative UFM1-specific protease, at least in normal conditions, is Ufsp2. This likely aligns with the analyses that Ufsp2 is much more abundant than Ufsp1 in many cells and tissues [30].

Detailed examination of the NMJ, a biological context where *Ufsp1* is highly expressed, revealed a minor structural alteration of the NMJ. Specifically, we observed a decreased overlap between the nerve terminals and the AChR clusters while the total area of AChR was unaffected. This finding somewhat contradicts our previous results in cultured cells, where loss of *Ufsp1* led to an increase in the number of AChR clusters [15]. This discrepancy likely reflects the complexity of NMJ biology *in vivo*, which involves interactions among different cell types. Indeed, the NMJ possesses a ‘safety factor’ that allows the synapse to maintain reliable neuromuscular transmission in different physiological conditions [3,33]. Thus, it is possible that in the *Ufsp1* KO/C53A the NMJ has adapted to reduce the number of synaptic vesicles (shown by synapsin staining in Fig 2) without altering the homeostasis of the synapse. Consequently, despite these subtle morphological changes, the loss of Ufsp1 or its enzymatic activity appears to be well-tolerated, with no observable impairments in muscle structure or function. While our targeted molecular profiling of major neurotrophic and NMJ factors did not reveal dysregulation in *Ufsp1* KO, unbiased approaches such as snRNA-Seq might uncover the molecular basis of these alterations in future studies. Notably, the fact that both of our mutants show the same NMJ morphological changes indicates that the enzymatic activity of Ufsp1 is required for this remodeling. Therefore, proteomic studies to identify UFMylated proteins in the presence and absence of Ufsp1 in mice or in C2C12 cells could provide insights into Ufsp1’s mechanism. Although our assessment is limited to select muscle types and a single time point, the data collectively point to a non-essential role for *Ufsp1* under baseline conditions. However, our results cannot exclude the possibility that Ufsp1 may impact the maturation kinetics of AChR clusters during embryonic and postnatal development.

While this study focused on skeletal muscle, it will be important to explore the role of Ufsp1 in other tissues, such as the brain and liver, where its expression is relatively high (Fig 1E). Additionally, given that that UFMylation is regulated by stress cues such as DNA damage, infection, and ER stress, it would be intriguing to investigate whether the role of Ufsp1 becomes apparent under pathological or challenged conditions, for instance ageing and exercise, in future studies. This is particularly noteworthy considering that active human Ufsp1 is translated from a non-canonical upstream start codon, a feature often associated with stress responses [34].

## Methods

### Generation of C53A line and genotyping

The Ufsp1 C53A knock-in line was obtained by the CRISPR/Cas9 approach. The Ufsp1 single exon (ENSMUSE00000384624) contains the Cysteine 53. We selected sgRNA using the CRISPOR web site (http://crispor.gi.ucsc.edu/) [35] with the following sequence: 5’-GGTAGCCGCATCCCCAGCCA-3’ (gR69, the sgRNA numeral refers to the MIT specificity score) and the single stranded donor DNA (ssODN) had the following sequence 5’-CAGGCCACTACCTTTACTATCACTATGGTTGCGATGGACTGGATGACCGTGGCTGGGGCGCCGGCTACCGCACCCTGCAGACGCTGTGCTCCTGGCCAGGGGGCCAGTCCTCGGGCGTGCC-3’. Three base substitutions from the wild type genomic sequence were introduced in this ssODN: two leading to the C53A mutation and the last one introducing a silent mutation as well as a new NarI restriction site for genotyping.

One hundred C57BL/6N fertilized oocytes were electroporated with a mix of cr/tracRNA (6 µM), wild type spCas9 protein (1.2 µM) and donor single stranded oligonucleotide (200 pmol/reaction). All microinjected eggs were reimplanted in B6/CBA foster females. Fifteen pups (putative founders) were born and analyzed by PCR. Three of them showed the presence of the NarI restriction site. The sequencing of the PCR product confirmed the presence of the C53A mutation. These founders were bred with WT mice, with which germ line transmission was achieved. A line (internal code Kus8481–2-PM; international nomenclature according to MGI: Ufsp1em1Ics) was established. Animals were housed in a controlled environment with a 12-h light/12-h dark cycle, with free access to water and a standard chow diet. Animal experiments utilizing Ufsp1 C53A mice were performed complying with the ARRIVE guidelines and in accordance with the regulations of Comite d’Ethique (animal ethics committee of IGBMC).

For genotyping the Ufsp1 C53A line, the primers F2- AGAAAACACCAGGAACCCGGAATTG and R1- CAGCCGATCCAGTTACGGGAGC were used with the annealing temperature of 62°C. PCR products were then digested with Nar1 enzyme for 1 hour at 37°C before checking their band lengths on agarose gel. Wildtype amplification product size is 338 bp, while amplification product size with the new Nar1 restriction site are 191 bp and 147 bp.

### Generation of KO line and genotyping

Ufsp1 KO allele was made via a dual sgRNA CRISPR/Cas9 approach. Briefly, two sgRNAs (target sequences: AGTAAAGGTAGTGGCCTGAG and CAGCAGCTTGTAGTTCACAA) were selected, according to the on- and off-target scores from the web tool CRISPOR (http://crispor.tefor.net) and chemically synthesized by IDT. To prepare the ribonucleoprotein complex (RNP) for each targeting, we incubated the sgRNA pair (50 ng/ul per sgRNA) and Cas9 protein (200 ng/ul; IDT) in Opti-MEM (Thermo Fisher) at 37°C for 15 min. The zygotes from superovulated female mice on the C57BL/6 genetic background were electroporated with 7.5 ul RNP on a glass slide electrode using the Genome Editor electroporator (BEX; 30V, 1ms width, and 5 pulses with 1s interval). Two minutes after electroporation, zygotes were moved into 500 ul cold M2 medium (Sigma), warmed up to room temperature, and then transferred into the oviductal ampulla of pseudopregnant CD-1 females. Pups were born and genotyped by PCR and Sanger sequencing. Animals were housed in a controlled environment with a 12-h light/12-h dark cycle, with free access to water and a standard chow diet. Animal experiments utilizing Ufsp1 KO mice were performed complying with the ARRIVE guidelines and in accordance with the regulations of the Institutional Animal Care and Use Committee-approved protocol of Cincinnati Children’s Hospital and Medical Center.

For genotyping the Ufsp1 KO line, the primers N1F-TGCCTCGATGACAGCTGCTCTAC, CR1- GATCTACCCAACGGTGCAGGTAG and WTR1- CCGCTCCTCTTCTCCCCTAAGC were used with the annealing temperature of 67°C. Wildtype amplification product sizes are 695 bp and 406 bp while amplification product size of the KO is a single band of 229 bp.

### In situ force measurement

In situ isometric muscle force measurement was performed as described previously [36]. Briefly, under anesthesia via isoflurane inhalation, the distal tendon of the TA was surgically exposed, cut, and attached to the lever arm (Aurora Scientific, 305C) by silk suture. Two electrodes were placed under the sciatic nerve, and electrical stimulation (0.2-ms pulse at 50 mA) was applied to elicit peak isometric twitch force at optimal muscle length. To determine peak isometric tetanic forces (Po), electrical frequency was increased from 12.5 to 150 Hz for 350 ms every 2 minutes. Immediately after the force measurement, mice were euthanized, and TA muscle weight and length were measured. The physiological cross-sectional area (PCSA) of TA muscle was estimated by the following equation: muscle mass (g)/(1.06 × muscle length [cm] × 0.6) [37]. Po was normalized to PCSA to calculate specific force. All data were collected and analyzed by Dynamic Muscle Control and Dynamic Muscle Analysis software (Aurora Scientific).

### Cross-sectional area measurement

The TA muscles were freshly dissected from mice in each cohort and embedded in OCT compound, frozen in 2-methylbutane chilled using liquid nitrogen and stored at −80°C for future use. For cross-sectional area analysis, the muscles were then cryo-sectioned with a thickness of 10µm. The muscle sections were fixed for 10 minutes with 4% PFA in PBS at room temperature. Following fixation, the sections were permeabilised with PBT washes (0.025% Tween-20 in PBS) for 30 minutes, and in PBX (0.5% Triton X-100 in PBS) for 6 minutes. After permeabilisation, the sections were blocked in 5% BSA/ 3% Horse serum/ 0.1% Triton X-100 in PBS for an hour in a humidity chamber before being incubated overnight in the rabbit anti-laminin antibody (Sigma-Aldrich; L9393) at 4°C. The washing step was then repeated with PBT, followed by incubation in the secondary antibody (Jackson ImmunoResearch) in the humidity chamber at room temperature for an hour. After the final washing step with PBT, the slides were mounted with Prolong™ Gold antifade reagent (Invitrogen), and representative images were taken on Confocal Leica SP8-UV. Cross-sectional area measurements were then calculated using ImageJ.

### Gripping strength measurement

To determine the grip strength, male mice aged 3-months from each cohort were placed on the grip grid (Bioseb Grip Test V3.22, Pinellas Park, FL, USA) and pulled gently backwards horizontally. The procedure was conducted on each mouse in triplicates and the maximal grip strength value was normalized to their body weight.

### Fiber type composition, stem cell number analysis and Gomori trichome staining

Transverse cryo-sections of 10µm thickness were first blocked in 3% BSA in PBS for 1 hour before incubating overnight at 4°C with the primary antibodies (1:20 Pax7, 1:50 BA-D5, 1:50 SC-71, 1:50 BF-F3- Developmental Studies Hybridoma Bank). Antigen retrieval for Pax7 was done as described previously [38]. Following primary antibody incubation, the sections were washed in PBS-T (0.1% Triton in PBS), prior to incubation with the secondary antibodies (1:1000 Goat anti mouse IgG1, Alexa Fluor 555, 1:100 IgG2b Cy3, 1:100 IgG1 488, 1:100 IgM DyLight 405- Jackson ImmunoResearch) and WGA (ThermoFisher; W32466) in blocking buffer for 1 hour at room temperature. The sections were then washed again in PBS-T before mounting. The representative images were taken on Confocal Leica SP8-UV. For quantification of Pax7^+^ stem cells and fiber typing, 10 images (40x) and 6 images (20x) per animal were analyzed, respectively, using Fiji [39]. For the Gomori trichome staining, 10µm thick sections were rehydrated through an alcohol series then stained with haematoxylin and Trichrome Stain LG Solution (HT10316; Sigma-Aldrich), following the protocol provided by the manufacturer.

### Western blotting

Dissected muscle tissues were snap frozen in liquid nitrogen and stored at −80˚C until use. For lysis, the frozen tissues were transferred into Precellysis tube (Tissue homogenizing CKMix – 2 mL; CAT. NO.: P000918-LYSK0-A) and ~300µl RIPA lysis buffer (50mM Tris-HCl with pH 7.5, 150mM NaCl, 2mM EDTA, 1mM MgCl_2_, 0.1% SDS, 0.1% sodium deoxycholate and 1% NP-40) was added. Tissues were coarsely chopped with surgical scissor. After incubating 10 minutes on ice, the tissues were lysed using Prcellysis homogenizer, and the same cycle was repeated once again. Tissue lysates were cleared by centrifugation on 13,000 rpm for 20 minutes on 4˚C. The supernatants were transferred to a new tube, protein concentration was measured by BCA assay (ThermoFisher), and boiled with 4X Laemmli buffer for 10 minutes (Bio-Rad). Tissue lysates were fractionated by 10% (for Ufsp1) or 8% (for the other two) SDS-PAGE gels and immunoblotted using the following antibodies: β-actin (Cell Signaling; #4970), Ufsp2 (Proteintech; # 16999–1-AP), and Ufsp1 (Sigma; HPA027099) – all in 1:1000 dilution in 5% BSA in TBS buffer with 0.1% Tween-20. For secondary antibody, we used anti-mouse and anti-rabbit HRP antibodies from Cell Signaling (#7076 and #7074). β-actin blot was obtained after stripping the Ufsp2 membrane using Western ReProbe™ PLUS (G Biosciences) according to manufacturer’s guideline.

### NMJ morphology analysis

Freshly isolated TA muscles were fixed in 4% PFA for 10 minutes, followed by washes in 1X PBS, then incubated overnight in 20% sucrose in PBS at 4°C. Following incubation, the muscles were embedded in OCT (Optimal cutting temperature) compound and frozen in 2-methylbutante in liquid nitrogen prior to sectioning. The samples were sectioned to a thickness of 40−50 µm. The muscle sections were first permeabilised with PBT for 30 minutes and blocked in 2.5% BSA/ 2.5% Goat serum in PBT for an hour. After the blocking, the samples were incubated overnight in 4°C with the primary antibodies; rabbit anti-neurofilament-L (1:500; Cell Signalling; #2837) and rabbit anti-synapsin-1 (1:500; Cell Signalling; #5297). Slides were washed in 1x PBS before incubation in a humidity chamber with secondary antibodies (Jackson ImmunoResearch) for an hour in room temperature. Mounting of the slides were done with Prolong™ Gold antifade reagent (Invitrogen). Quantification of the areas and overlap were calculated through the aNMJ-morph macro workflow [40] on Image J.

### RNA isolation and RT-qPCR

Total RNA was extracted and isolated from flash frozen gastrocnemius (GA) muscles using Trizol reagent (Molecular research centre; TR118), and homogenized in the Precellys evolution machine using Precellys tubes from Precellys lysing kit (Precellys; P000918-LYSK0-A). The cDNA synthesis was then conducted using the SuperScript™ IV Reverse Transcriptase (ThermoFisher; 18090010) with a combination of Oligo (dT) 18 primer (ThermoFisher; #S0142) and random Hexamer Primer (ThermoFisher; #S0132). The qPCR analysis was done on a LightCycler® 480 Instrument II (Roche) using FastStart SyberGreen master mix (Roche). The primers used in this study are as follows. *Ngf* F: 5’-TCCACCCACCCAGTCTTC-3’, *Ngf* R: 5’-GCTCGGCACTTGGTCTCA −3’, *Bdnf* F: 5’-AAGGACGCGGACTTGTACAC-3’, *Bdnf* R: 5’-CGCTAATACTGTCACACACGC-3’, *Nt3* F: 5’-TAAAGAAGCCAGGCCAGTCA-3’, *Nt3* R: 5’-AGTCAGTGCTCGGACGTAGG-3’, *Chrna* F: 5’-CTTAACCAGCCTGGTGTTCTACC-3’, *Chrna* R: 5’-GCTCCACAATGACCAGAAGGAAC-3’, *Chrnb* F: 5’-TATTCGGCGGAAGCCTCTCTTC-3’, *Chrnb* R: 5’-GCAGCAAGAACACAGTGAGCGT-3’, *Chrnd* F: 5’-CACACTTAGCCTGAAGCAGGAG-3’, *Chrnd* R: 5’-GGTCCACATTGAGCTTGGCTGC-3’, *Chrne* F: 5’-CACACTTAGCCTGAAGCAGGAG-3’, *Chrne* R: 5’-GGTCCACATTGAGCTTGGCTGC-3’, *Colq* F: 5’-CAAGCTCTTCCTGGTTGTTGACC-3’, *Colq* R: 5’-CCTCCAGGAAGATGCCTTTGCG-3’, *Musk* F: 5’-TGAAGCTGGAAGTGGAGGTTTT-3’, *Musk* R: 5’-GCAGTAGGGTTACAAAGGAA-3’

### Animal experiments

Mice were housed in constant ambient temperature (23 °C), humidity (56%), and light-night cycle (light on 6:00 am and light off 6:00 pm). The animals were euthanized by a gradual increase of CO2 inhalation (to 100%) across a 3 min interval. Male mice were used for all experiments, which were performed at 3 months of age. Although our mutants did not produce any harmful phenotype, the health of animals were constantly surveyed by our institutions’ animal caretakers and veterinarians to ensure their welfare and minimize any type of suffering.

### Statistical tests

Statistical analysis was conducted using GraphPad Prism 8 software and regarded significant when p-values were lower than 0.05. No data points were regarded as outliers nor removed from the analysis. The detailed tests and n numbers are indicated in each figure legend.

## Supporting information

S1 Fig Normal skeletal muscle architecture in Ufsp1 C53A mutant. **(A)** Tibialis anterior (TA) and gastrocnemius (GA) muscle weights were measured (n = 3). **(B-C)** Cross-sectional areas (CSA) of TA and Soleus muscles were quantified based on ColV immunohistochemistry (n = 3). Scale bar, 100 µm. Error bars indicate S.E.M. Two-tailed, unpaired student’s t-test. ns, non-significant.
(TIF)

S2 Fig Characterization of fiber types, degeneration, stem cell and mitochondria in *Ufsp1* KO muscles. **(A)** Representative images of fiber type immunohistochemistry in WT and *Ufsp1* KO TA muscles and quantification of each fiber type distribution (n = 3). **(B)** Same as (A) for soleus muscles (n = 3). **(C)** Representative images of Pax7 (stem cell) and Dystrophin (muscle membrane) immunostaining in WT and *Ufsp1* KO TA muscles. Frequency of Pax7-positive cells (per 100 myofibers) and centralized myonuclei were quantified from these images (n = 3). **(D)** Same as (C) for soleus muscles (n = 3). **(E)** Representative images of Gomori trichome staining in WT and *Ufsp1* KO muscles (TA and soleus). 3 animals per condition were stained with the same results. Error bars indicate S.E.M. ns, non-significant. Two-tailed, unpaired t-test.
(TIF)

S3 Fig Characterization of muscle output and NMJ gene expression profile in *Ufsp1* KO muscles. **(A)** Grip strengths were measured for 10-weeks old male control and mutants (n = 4). **(B)** Peak isometric tetanic force (P_o_), peak isometric twitch force (P_t_) and the rate of relaxation during P_o_ of TA muscle were measured in situ condition and compared between WT and KO (n = 3). **(C)** RT-qPCR of indicated genes in TA and GA muscles (n = 3). Error bars indicate S.E.M. ns, non-significant. Two-tailed, unpaired t-test.
(TIF)

S1 File Raw data. (XLSX)

S2 File Raw images. (PDF)
