# Supplementary figures and images for: Loss of Ufsp1 does not cause major changes at the neuromuscular junction
Source: PLoS One. 2025 Aug 1;20(8):e0328690. doi: 10.1371/journal.pone.0328690 (PMC12316231; doi:10.1371/journal.pone.0328690)

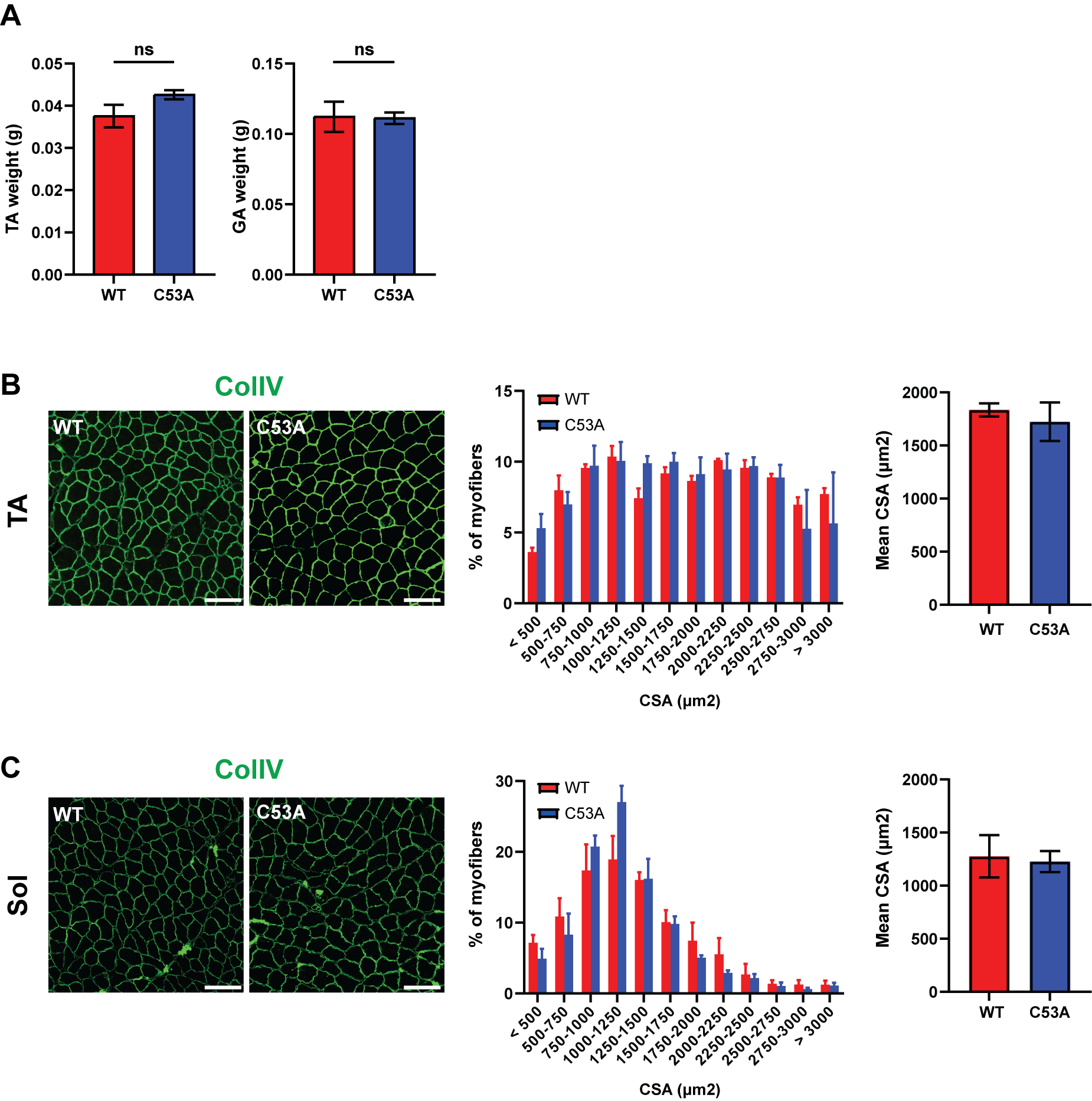

Supplement: S1 Fig — (A) Tibialis anterior (TA) and gastrocnemius (GA) muscle weights were measured (n = 3). (B-C) Cross-sectional areas (CSA) of TA and Soleus muscles were quantified based on ColV immunohistochemistry (n = 3). Scale bar, 100 µm. Error bars indicate S.E.M. Two-tailed, unpaired student’s t-test. ns, non-significant. (TIF) [file pone.0328690.s001.tif]

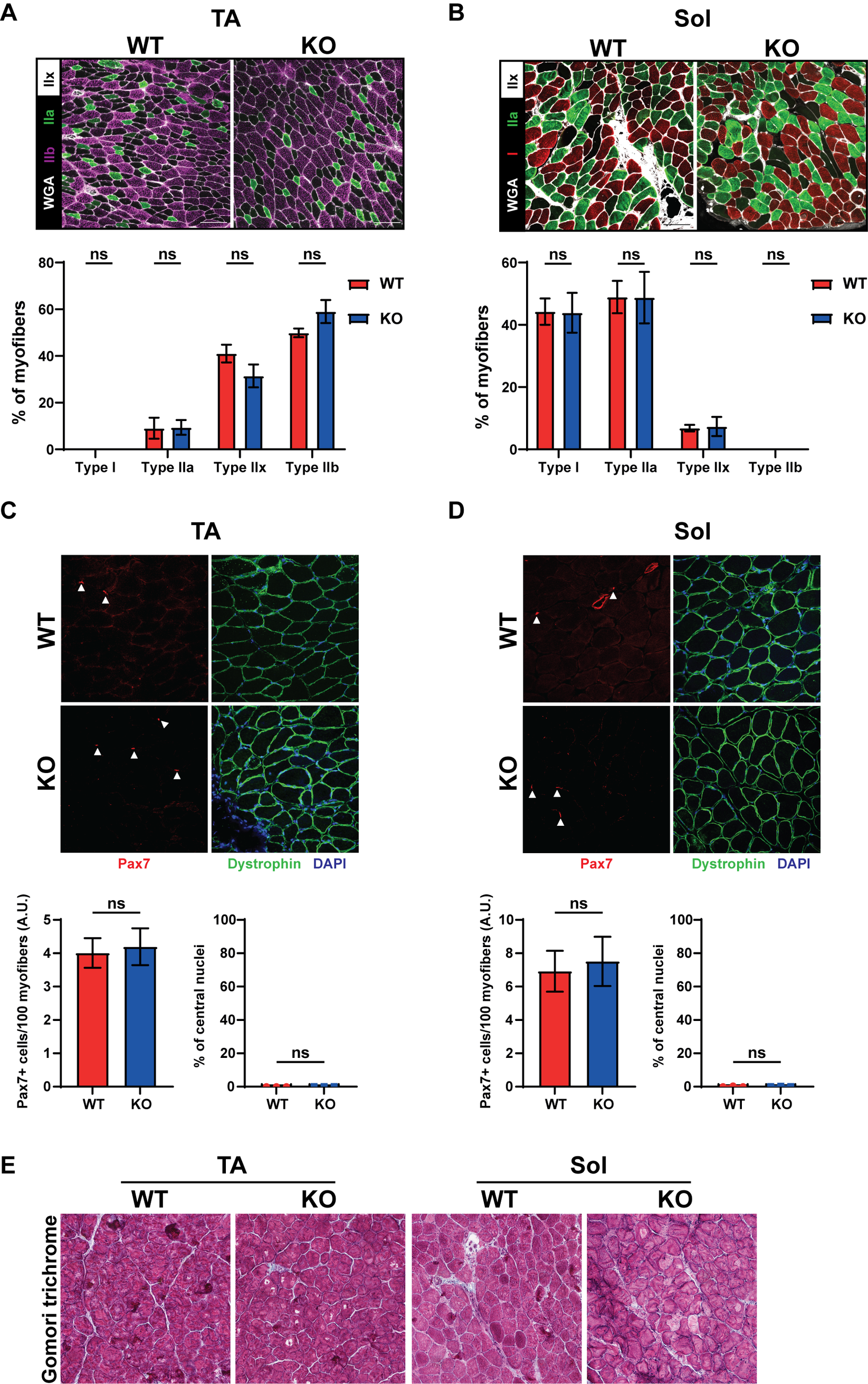

Supplement: S2 Fig — (A) Representative images of fiber type immunohistochemistry in WT and Ufsp1 KO TA muscles and quantification of each fiber type distribution (n = 3). (B) Same as (A) for soleus muscles (n = 3). (C) Representative images of Pax7 (stem cell) and Dystrophin (muscle membrane) immunostaining in WT and Ufsp1 KO TA muscles. Frequency of Pax7-positive cells (per 100 myofibers) and centralized myonuclei were quantified from these images (n = 3). (D) Same as (C) for soleus muscles (n = 3). (E) Representative images of Gomori trichome staining in WT and Ufsp1 KO muscles (TA and soleus). 3 animals per condition were stained with the same results. Error bars indicate S.E.M. ns, non-significant. Two-tailed, unpaired t-test. (TIF) [file pone.0328690.s002.tif]

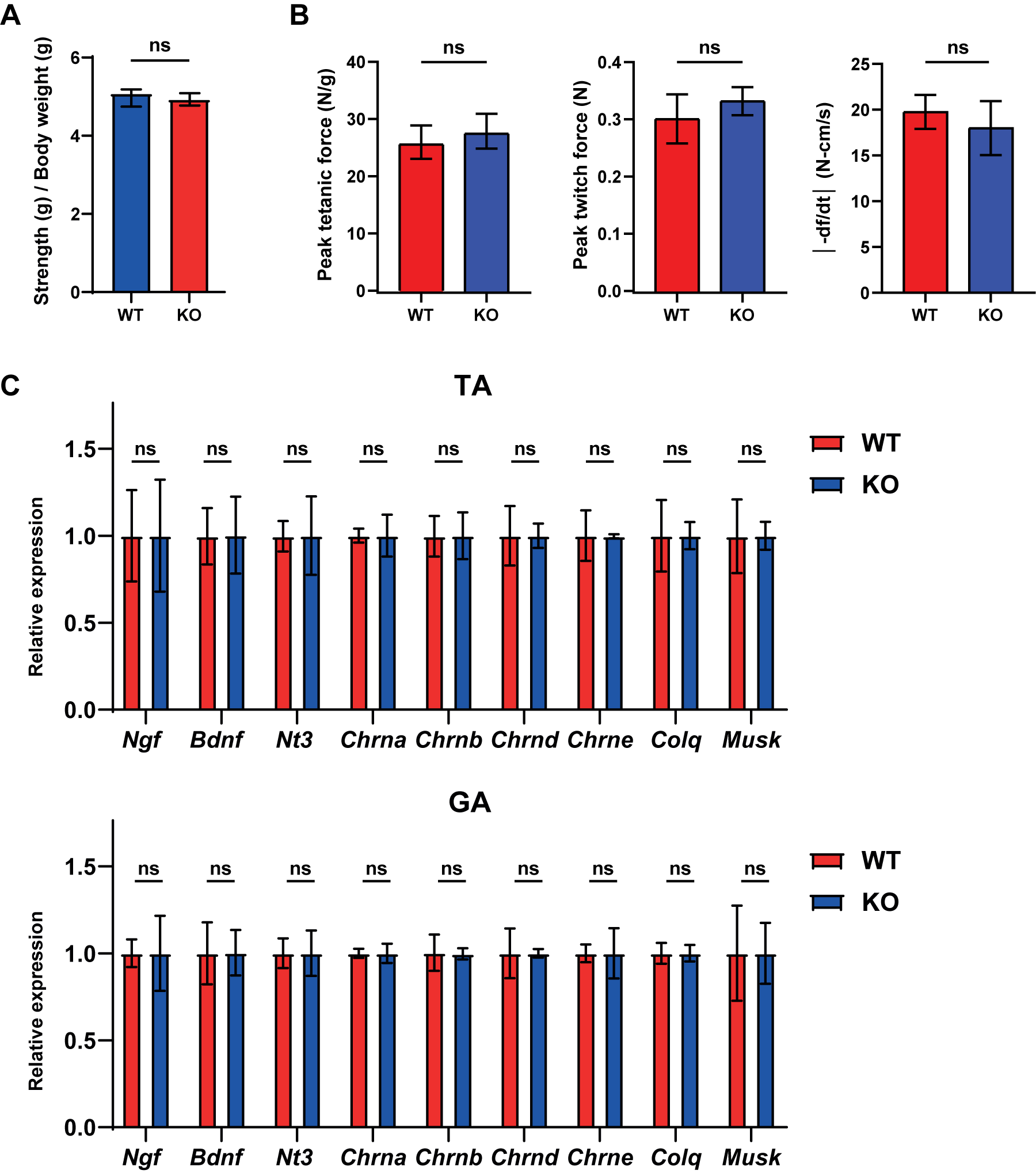

Supplement: S3 Fig — (A) Grip strengths were measured for 10-weeks old male control and mutants (n = 4). (B) Peak isometric tetanic force (Po), peak isometric twitch force (Pt) and the rate of relaxation during Po of TA muscle were measured in situ condition and compared between WT and KO (n = 3). (C) RT-qPCR of indicated genes in TA and GA muscles (n = 3). Error bars indicate S.E.M. ns, non-significant. Two-tailed, unpaired t-test. (TIF) [file pone.0328690.s003.tif]

Figure 1B. Genotyping of Ufsp1 CA

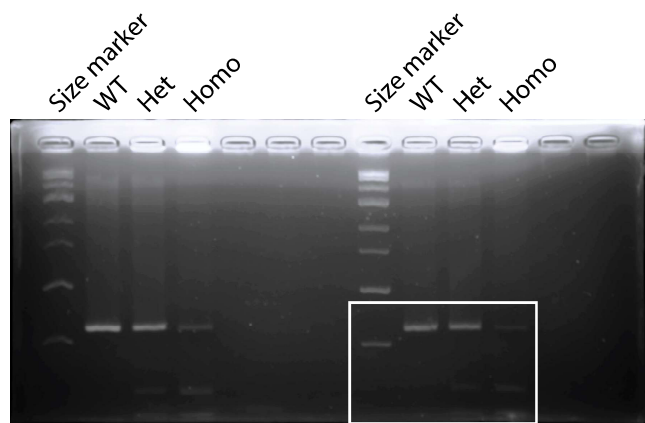

Figure 2A. Western blot validation of Ufsp1 KO

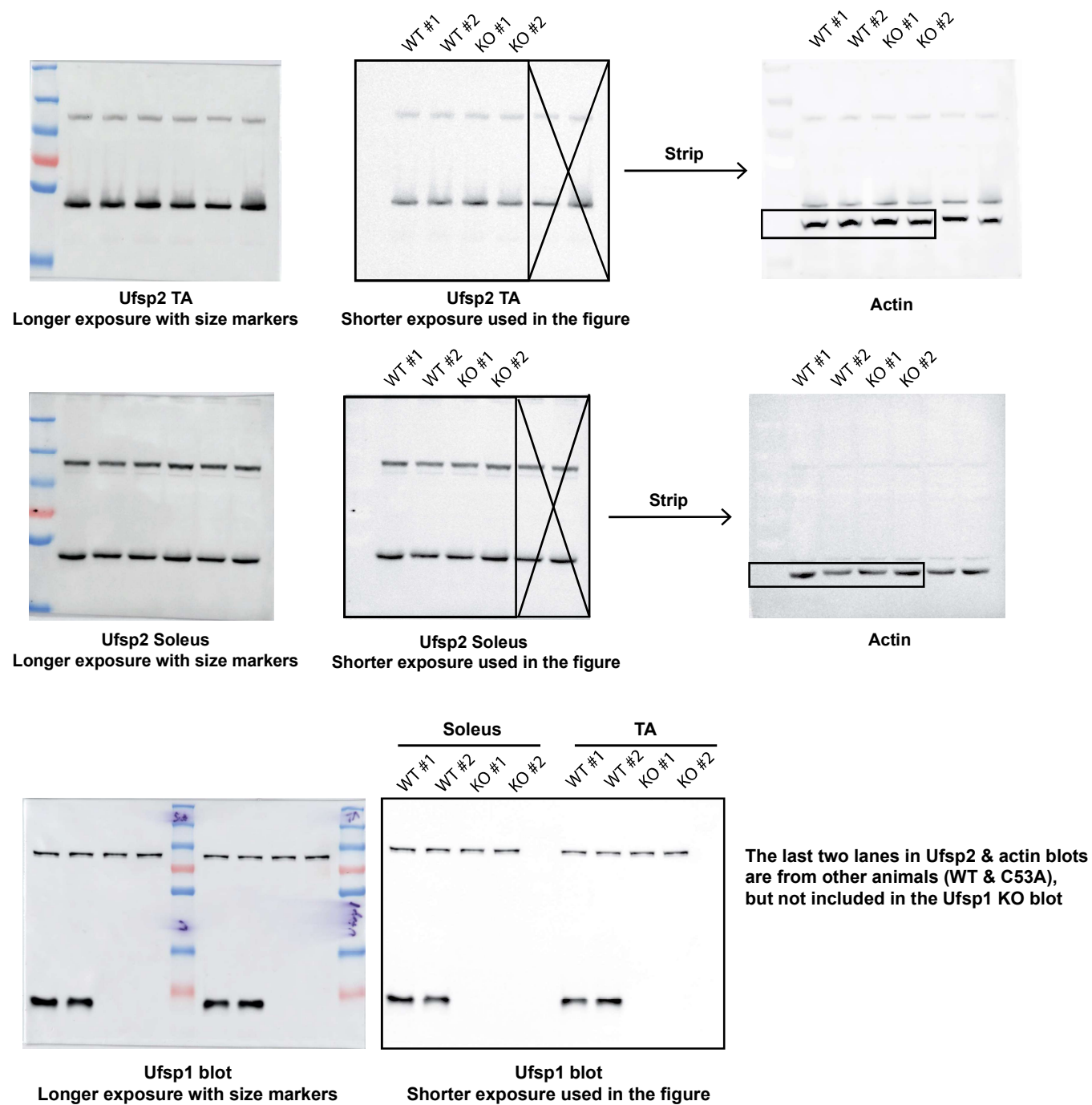

Supplement: S2 File — (PDF) [file pone.0328690.s005.pdf]
